# High-performance computing for SARS-CoV-2 RNAs clustering: a data science‒based genomics approach

**DOI:** 10.5808/gi.21056

**Published:** 2021-12-31

**Authors:** Anas Oujja, Mohamed Riduan Abid, Jaouad Boumhidi, Safae Bourhnane, Asmaa Mourhir, Fatima Merchant, Driss Benhaddou

**Affiliations:** 1 School of Science and Engineering, Al Akhawayn University in Ifrane, Ifrane 53000, Morocco; 2 Computer Science, Signals, Automation and Cognitivism Laboratory (LISAC), Computer Science Department, Faculty of Science Dhar El Mahraz, Sidi Mohamed Ben Abdellah University, Fez 30000, Morocco; 3 Faculty of Sciences, Chouaib Doukkali University, El Jadida 24000, Morocco; 4 Computer Engineering Technology Faculty, University of Houston, Houston, TX 77204, USA

**Keywords:** bioinformatics, data science, high-performance computing, longest common subsequence, RNA, SARS-CoV-2

## Abstract

Nowadays, Genomic data constitutes one of the fastest growing datasets in the world. As of 2025, it is supposed to become the fourth largest source of Big Data, and thus mandating adequate high-performance computing (HPC) platform for processing. With the latest unprecedented and unpredictable mutations in severe acute respiratory syndrome coronavirus 2 (SARS-CoV-2), the research community is in crucial need for ICT tools to process SARS-CoV-2 RNA data, e.g., by classifying it (i.e., clustering) and thus assisting in tracking virus mutations and predict future ones. In this paper, we are presenting an HPC-based SARS-CoV-2 RNAs clustering tool. We are adopting a data science approach, from data collection, through analysis, to visualization. In the analysis step, we present how our clustering approach leverages on HPC and the longest common subsequence (LCS) algorithm. The approach uses the Hadoop MapReduce programming paradigm and adapts the LCS algorithm in order to efficiently compute the length of the LCS for each pair of SARS-CoV-2 RNA sequences. The latter are extracted from the U.S. National Center for Biotechnology Information (NCBI) Virus repository. The computed LCS lengths are used to measure the dissimilarities between RNA sequences in order to work out existing clusters. In addition to that, we present a comparative study of the LCS algorithm performance based on variable workloads and different numbers of Hadoop worker nodes.

## Introduction

In 2003, the world manifested an unprecedented breakthrough by completing the first full human genome sequencing in history. It took 13 years and costed $2.7 billion [1]. Since then, the cost and time to sequence genomes have decreased significantly which led to an increase in the number of sequenced genomes. This allowed researchers in many fields to better answer biological questions by using a set of recently developed computational tools to address problems that were hard or impossible to solve for many decades. However, the continuous increase of generated data has imposed growing difficulties in analyzing them.

Nowadays, Genomic data is one of the fastest growing big datasets in the world, and as of 2025 it is supposed to become the fourth largest source of Big Data after Astronomy, YouTube, and Twitter [2]. Numerous genomes of many species are widely sequenced and stored in public online Gene Banks. These Gene Banks are provided by many biology institutes like the National Institute of Health (NIH) in the United States [3] and the National Institute of Genetics (NIG) in Japan [4].

This work emerges within the context of the current coronavirus disease 2019 pandemic. Thus, it is principally meant to contribute to understanding severe acute respiratory syndrome coronavirus 2 (SARS-CoV-2) mutation behavior. Therefore, limiting its spread all over the world, by providing a genomics tool that will answer relevant questions regarding SARS-COV-2 virus genomic evolution.

SARS-CoV-2 is a positive-sense single-stranded RNA virus which keeps mutating in an unpredictable way. We can find a large number of SARS-CoV-2 RNA sequences stored in NCBI Virus repository which is accessible to the public through a simple internet connection [5].

Clustering SARS-CoV-2 sequences and the classification of the different variations of the virus is crucial to demystifying its unpredictable mutations and thus limiting its spread. The virus medical treatment, and confinement policies, might tangibly differ from a village/city/region/country to another depending on the residing SARS-CoV-2 virus type. Reinforced by the early confinement and travel restriction laws, the SARS-CoV-2 viruses are very likely to be limited in the number of types. However, this does not exclude the probability of having virus mutations that happened locally in each village/city/region/country as well. In this project, we are identifying the existing SARS-CoV-2 virus types and track back the mutations that have happened or are still happening. In fact, clustering is a data mining method that aims to identify similar groups in huge datasets, and is widely used in various bioinformatics fields, such as cancer class discovery [6] and protein structure prediction [7].

Belonging to the realm of Big Data, SARS-CoV-2 RNA sequences mandates high-performance computing (HPC) for processing. Furthermore, we need optimal efficient algorithms and approaches to measure similarities between each pair of RNAs. In this context, we developed a distributed approach, based on the Hadoop framework and started comparing SARS-CoV-2 RNA sequences by using the longest common subsequence (LCS) algorithm to finally measure similarities between sequences and build relevant clusters. Computing the LCS consists of finding the longest subsequence common to two sequences. LCS algorithm leverages dynamic programming (DP) to simplify the problem by breaking it down into simpler sub-problems in a recursive manner. In bioinformatics, DP is commonly used for tasks like sequence alignment, protein folding, RNA structure prediction, and protein-DNA binding [8,9].

To prove the indispensability of HPC for RNA sequences processing, we run extensive experimentations on a Hadoop cluster. We used variable Hadoop worker node sets along with a variable workload. The latter consisted of varying the number of pairwise comparisons between the different collected RNA sequences. Both used data and obtained results are made open and available online [10].

Data science (DS) is considered a novel and very promising science that combines statistics, data analysis, informatics, and related methods to better understand and analyze actual ‘phenomena' involving large amounts of data [11]. It is an interdisciplinary field that focuses on extracting knowledge from large data sets and applying that knowledge and actionable insights to solve problems.

There are five principal DS steps that should be performed correctly to reach the objective of this work:

‒ Acquisition: Acquiring SARS-CoV-2 RNA sequences
‒ Preparing: It consists of exploring and pre-processing data in such a way that only valid and adequate data are kept for appropriate analysis.
‒ Analyze: This is the step where we are leveraging on acquired skills in HPC, and distributed computing in general, to optimize the processing. The analysis in this work is divided into three steps that we are detailing later in this paper.
‒ Report: Once the analysis is done, the remaining challenging problem would be to visually present the results in an efficient and readable way to explain to the action makers what should be done to improve the results in terms of biological value.
‒ Action: After presenting the blueprint for an open-source HPC and LCS-based platform for SARS RNA clustering. This can help researchers in medicine and virologist in clustering and identifying the rapid variance in SARS-CoV-2.

## Main Text

### Sequence alignment

In order to measure the similarity between genomic sequences despite their nature, we are opting for the most conventional approach that uses sequence alignment techniques. Sequence alignment is a daily task of most biologists in order to find the relationship or similarity between biological sequences. In bioinformatics, a sequence alignment is considered a way of arranging the sequences of DNA, RNA, or protein to identify regions of similarity that may be a consequence of functional, structural, or evolutionary relationships between the sequences [12]. Most of the interesting genomic problems require the alignment of lengthy sequences with numerous variations.

There are two categories of alignment; global alignment and local alignment. Global alignment assumes that the two sequences to align are closely related and of the same length. The alignment is carried out from the beginning until the end of the sequence. A general algorithm to perform global alignment is the Needleman-Wunsch algorithm [13]. Sequences which are suspected to have similar or even dissimilar subsequences can be compared using local alignment method. This method finds local regions with a high level of similarity. A general algorithm to perform local alignment is the Smith-Waterman algorithm [14].

Needleman-Wunsch alignment is based on evaluating the collinearity of two amino acid or nucleotide sequences; it considers a global alignment of the sequences and works optimally when comparing highly similar sequences. The Smith-Waterman algorithm considers local alignments of similar regions that fall within what may be dissimilar sets of sequences. Both algorithms can be too simplistic thus slow for large sequences. Nevertheless, the exact local and global alignments are those returned by Smith-Waterman and Needleman-Wunsch algorithms, respectively.

Because of the slowness of conventional algorithms aforementioned, many alignment tools have been developed to address that problem. The vast majority of algorithms used by the bioinformaticians nowadays borrow from the concepts of either or both of the Needleman-Wunsch and Smith-Waterman algorithms with the only advantage of accelerating the processing time. BLAST (Basic Local Alignment Search Tool) [15] is the most popular local alignment program for similarity search and sequence alignment for large datasets. The BLAST algorithm is a heuristic algorithm. It generates a list of short word matches (default word size is 11 for nucleotide) in query sequence. The database is then searched for the occurrences of these words. The matching words are extended to the local alignment between two sequences and these extensions are continued until the score is below a threshold. Another local alignment tool is the FASTA program developed by Pearson and Lipman [16]. FASTA searches for short sequences called k-tuples (which are similar to words in BLAST) to identify un-gapped alignments. The alignments are tested and merged into a local alignment in order to find the optimal local alignment based on the threshold and score. FASTA provides tools similar to BLAST. However, it also performs global alignments which are not provided by BLAST.

BLAST and FASTA are based on heuristic algorithms, and due to the huge volume of produced data, it is impossible to process all data in only one computer accurately. It is then mandated to use an appropriate approach along with an accurate algorithm to measure similarities between large sequences. Thus, LCS would be the most appropriate approach to compute similarity between a pair of sequences since it is combining both approaches, i.e., local and global alignment. Neither, the lengths of both sequences and precision will be a constraint to respect.

### Problem statement

Regardless of the field on which clustering is used, it is known that all clustering algorithms are either implicitly or explicitly oriented by a variety of similarity measures that quantifies the distinctness of each pair of elements. The majority of clustering algorithms used in the literature require considering the sequences (variables) as a set of parameters that are numerical values in order to compute the similarity using distance algorithms. Thus, for genomic sequences clustering, this is not the best option to go for since it will lead to extending the processing time due to the large number of nucleotides or amino acids present in the sequences. Also, and even if features should be extracted, there is no clue what features should be involved to better characterize the sequences. Then, another step will be needed to look for the most relevant features among all extracted features which will add more time to the process.

In genomics, very similar to the alignment algorithms, the LCS algorithm is used to measure dissimilarity between sequences. Moreover, it has shown efficient results in compressing sequences of DNA [17]. Computing the length of the LCS between a pair of sequences along with using an appropriate similarity measure would be an alternative to get rid of the step of extracting features and get accurate results of clustered SARS-CoV-2 sequences.

The great number of sequences needed to achieve the ultimate goal of this work, and the nature of the SARS-CoV-2 itself whose size varies from 26K to 32K nucleotides implies another challenge that resides in the need to store sequences and process them in a reasonable time. The sequences’ data falls within the realm of Big Data. In fact, SARS-CoV-2 is a virus which keeps mutating all the time, and so far, there are more than 48.000 SARS-CoV-2 RNAs stored in NCBI Virus repository submitted from all over the world [18].

We have noted in our primary experiment, performed on an ordinary laptop, that the time to run a simple pairwise comparison using the LCS between sequences is around 9 s. To compare all n RNA sequences among each other, i.e., a set-comparison, we need (n * (n-1)/2) single pairwise comparisons: with 100 RNAs, and with 9 seconds for each comparison, the total needed time becomes (100 × 99)/2 × 9 (s) = 12 h approximately. With 10.000 samples, we would need 14 y. This is where HPC comes into play and proves indispensable to optimize the execution time.

### HPC infrastructure

Nowadays, biological data is exploding into petabytes, necessitating HPC to assemble a variety of genomic datasets and compare them. HPC has been around since the dawn of computing, and it was used to solve and analyze complex problems requiring substantial compute power in terms of both processing and storage [19-22].

In this work, we deployed Hadoop on a cluster of five computers to store the data consisted of all SARS-CoV-2 sequence pairs which are then processed by computing the length of the LCS.

#### Hardware

The use of HPC capabilities proves crucial to address the problem of high execution time. For our HPC infrastructure, we opted for the most appropriate hardware resources for storing, computing, and communicating data efficiently.

We built our infrastructure using five computers on which data should be distributed in the form of data chunks which are small data portions of possible sequence pairs in order to get processed in parallel. Each data chunk is locally stored in at least one computer that is responsible for its processing. This is done to optimize the execution time. All the data needed for this work are retrieved from the NCBI Virus repository on a single computer. This computer does not perform any computations except to distribute and collect data via the network. It is called the Master.

The Master and all other computers (the Slaves) that are responsible for processing data have 8 GB memory capacity, and the communication between the Master and all Slaves is possible by using a switch with a connection speed that goes up to 1,000 Mbp.

#### Hadoop deployment

**Fig. 1.**
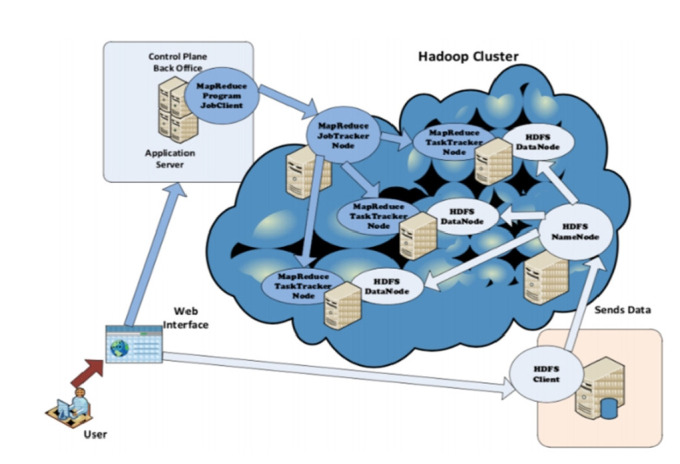
The general architecture of high-performance computing service in a private datacenter using Hadoop [25].

Our HPC infrastructure consists of five computers linked through a network on which Hadoop [23] is deployed in each machine. This gives the ability to store genomic Big Data through Hadoop’s distributed file system (HDFS) among different nodes, and to process data using the well-known MapReduce [24] programming paradigm that runs the “jobs” on selected chunks of Big Data, see Fig. 1.

HDFS contains two kinds of nodes:

‒ *Namenode*, which is the master node whose task is to manage the distributed file system by keeping relevant metadata and namespace entries.
‒ *Datanodes (workers)*, which store and extract blocks upon requests from the Namenode.

On the other hand, we have the MapReduce component that takes care of the processing of the Big Data stored in the HDFS. This involves three main entities:

‒ *JobTracker*: Coordinates job execution by splitting the main job into tasks, and delegates them to the TaskTrackers while considering two essential factors: load balancing and location of the chunks in the HDFS Datanodes.
‒ *TaskTracker*: The MapReduce horse-workers that run the tasks assigned by the JobTracker.
‒ *HDFS*: Responsible for providing the chunks to the TaskTrackers.

To distribute algorithms/programs (e.g., using MapReduce) among clustered CPUs, the program needs to be “distributable.” For instance, to compute the average of a large set of numbers (e.g., 1 trillion numbers), we can use a cluster of 10 nodes and divide (i.e., map) the 1 trillion set of numbers equally among the different nodes. Then, every node will separately compute its (local) average and report the result back in order to compute the grand average. This way, we leveraged HPC by having 10 CPUs working in parallel to perform a time-consuming task, alike the inherent parallel processing inside a single super-computer where CPUs are sharing both the memory and the clock. Unfortunately, not all algorithms are “distributable”: If we take the well-known “shortest-path” algorithm on graphs. We cannot divide (Map) the Graph into smaller graphs and distribute them among nodes. It is unfeasible as the answer to “shortest path?” would involve treating the whole input/graph as a single entity.

Similarly, in genomics, when comparing two DNAs/RNAs, the latter should not be dissected into parts and then distributed among the different clustered nodes to do the comparison. Fortunately, the SARS-CoV-2 RNAs are far less in terms of size than human DNAs: 30 kB vs. 3 GB, respectively. This corresponds to an order of magnitude of 0.0001%. However, to run a comparison between hundreds of RNAs, HPC proves indispensable as the comparison follows a polynomial growth of O(n^2^).

### Proposed approach

In this section, we are presenting the proposed approach to cluster SARS-CoV-2 sequences and describe all steps needed to reach the final objective of clustering SARS-CoV-2 sequences.

Genomics value chain (GVC) is the conventional pipeline to process genomic data. It delineates the different steps from biological substances (e.g., blood, saliva, etc.) to useful information that can be applied in curing diseases or improving the health of citizens.

**Fig. 2.**
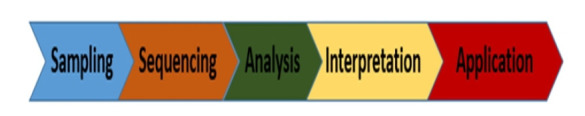
Genomics value chain. (1) Sampling: collecting DNA/RNAs source, (2) Sequencing: generating the order of the nucleotides (A, T, C, G) in the DNA/RNA, (3) Analysis: compute dissimilarity between sequences using longest common subsequence algorithm, (4) Interpretation: translating observed results into knowledge, (5) Application: proposing solutions.

GVC consist of five stages as shown in Fig. 2.

In this work, the Sampling and Sequencing steps are already carried out by NCBI, and data is openly available.

We are leveraging on acquired skills in HPC, and distributed computing in general, to optimize the GVC analysis step (3). Then, after successfully carrying out the Analysis step where clustering is performed, the other main remaining challenge is step-4 (i.e., Interpretation). This consists of visualizing the results in a readable way in order to well understand them, and this is where specialists in immunology, biology and medicine should play a decisive role in confirming its relevance to our target goal and propose solutions in the Application step.

Nowadays, researchers are interested in what DS has to offer in the field of bioinformatics, as in any other fields with access to large data sets. In fact, DS details the GVC especially when working with large data sets in which data is not totally valid and should get prepared for analysis. After collecting more than 6.000 SARS-CoV-2 sequences from NCBI Virus repository and we tweaked our DP algorithm for computing the lengths of LCS using the Tabulation approach.

In fact, there are two different ways to store the values of a computation so that the values of a sub-problem can be reused. Both approaches conclude to the same result, the difference simply lies in the way of conveying the message and that’s exactly what Bottom-Up (Tabulation) and Top-Down (Memoization) DP do. For Bottom-Up approach, as the name itself suggests starting from the bottom (first position) and accumulating answers to the top destination state (the whole sequence). In contrast, for Top-Down approach, we begin at the topmost destination state and work our way down to the bottommost base state, counting the values of states that can reach the destination state. We used Tabulation instead of Memoization as Tabulation shows less processing time compared to Memoization. The outputs of the LCS algorithm serve then to compute dissimilarity between each pair of sequences in the dataset and which in return are used to build clusters on which each cluster would contain similar sequences.

#### DS step 1: data retrieving

It is the first step of the process. We collected 6.326 SARS-CoV-2 RNA sequences from NCBI Virus repository for the matter of this work. The first observation that we noticed concerning the dataset is that sequences are of different sizes; ranging from 20 nucleotides to 30K nucleotides.

#### DS step 2: data preparing

In fact, this step splits into two complementary steps that are necessary to perform before moving towards analysis. First, the nature of the studied genomic data must be known the best possible as well as its properties and characteristics. Therefore, we proceed to know the nature of data by performing some research. This confirmed that the SARS-CoV-2 RNA sequence length ranges from 26K to 32K bases and that the GC-content, which is a good scale to measure the correctness of structure data by itself, is around 38% [26,27]. The second sub-step is the process that complements the knowledge of the studied data nature. It is data cleansing in which all erroneous and irrelevant data are removed from the data set. Indeed, all data that are far from having a GC-content around 38% and sequences that are not ranging from 26K to 32K in length are removed. Sequences that show incertitude during sequencing are removed as well. Thus, out of 6.326 sequences, only 41.6% that corresponds to 2.635 sequences may be processed.

Therefore, 2.635 sequences can be used in this work without worrying about working with erroneous data. During this study, we limited ourselves to 120 RNAs from the collected SARS-CoV-2 sequences and tweaked our DP algorithm to use Tabulation running the LCS algorithm along with the proposed distance and distance algorithms.

The great number of removed sequences that are stored in the NCBI database (approximately 58.4%) is due to its submission-free policy. Thus, any entity in the world has the ability to submit whatever sequence without restrictions or checking by NCBI.

#### DS step 3: analysis

**Fig. 3.**
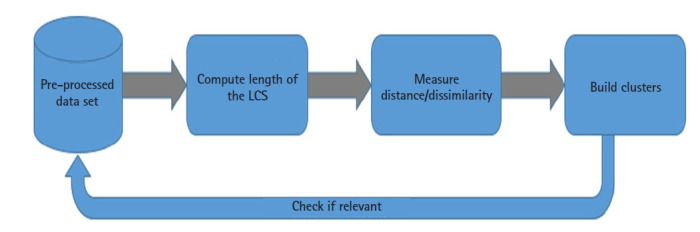
Analysis flowchart. LCS, longest common subsequence.

The analysis is the main focus of this work. The final objective is to cluster SARS-CoV-2 sequences by computing the lengths of LCSs and measure dissimilarity based on LCSs lengths for each pair of sequences. The analysis flowchart we adopted in this work is illustrated in is Fig. 3.

After collecting and cleansing data, we start analyzing sequences. The analysis begins by performing comparisons between each pair of sequences in the dataset using the LCS algorithm. Then, measure dissimilarity to eventually cluster the sequences. Once all the sequences get clustered, the relevance of the clusters to the properties of each sequence should be checked and used as a performance metric for the proposed methodology.

##### Length of LCS computing

There are two different approaches used conventionally in bioinformatics to measure similarities over a dataset of nucleotide sequences, that are local and global alignments. Calculating a global alignment is a type of global optimization that requires the alignment to span the complete length of the query sequences while also requiring that the sequences be of the same length. Local alignments, on the other hand, detect similar sections within long sequences that are typically highly diverse overall. Local alignments are generally preferred, but they can be more difficult to calculate due to the additional difficulty of locating similarity regions [28]. Even though, some heuristic/probabilistic methods have been designed for large-scale database search; they do not guarantee to find best matches [15,16]. Then, computing LCS came to be a good choice to measure similarity since it does not show any restrictions regarding the sequence’s length when comparing a pair of sequences as a whole and is accurate as well when used next to an appropriate distance measurement formula.

The LCS problem is a classical problem in computer science. Given two strings X and Y, the LCS problem is to find the longest subsequence common to both X and Y. In genomics, very similar to the alignment algorithms, the LCS algorithm is used to measure dissimilarity between sequences. Moreover, it has shown efficient results in compressing sequences of DNA [17], which is of major utility when large data needs to be shared or stored in limited storage resources. Tabulation is an approach where we solve a problem by first filling up a table, and then computing the solution to the original problem based on the previous results in this table.

All possible pairs of characters are represented in a 2D matrix. The sequences are written across the top and down the left side of the matrix, except that an extra row and column are added in the first position of the matrix to allow the process to begin. The overall computation of LCSs lengths is performed using the MapReduce programming paradigm where each worker is responsible for the mapping of a subset of sequence pairs to their corresponding LCS lengths.

**Fig. 4.**
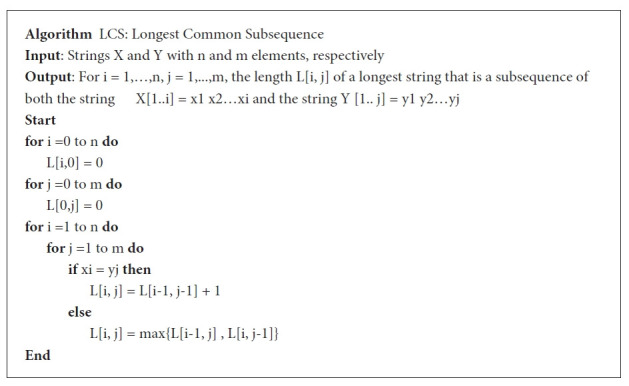
Longest common subsequence (LCS) algorithm.

Following is the algorithm that we are adapting to compute the length of the LCS of each pair of SARS-CoV-2 sequences (Fig. 4).

L (n, m) is the length of the LCS, and it is a parameter we are using in addition to the lengths of both sequences X and Y to compute the dissimilarity between the sequences.

We did implement the algorithm using an ordinary laptop and we obtained the results presented in Table 1.

From Table 1, we note that the time it took to run a hundred pairwise comparisons is 14 minutes approximately. This means that it takes around 9 s to finish one single comparison on average. To compare n RNA sequences among each other, i.e., a set-comparison, we need (n * (n‒1)/2) single pairwise comparisons:

‒ With 30 RNAs, we performed (30 * 29)/2 = 435 comparisons and it took about 59 min to finish.
‒ With 2.635 samples, we would need about 1 y and this is where HPC comes into play and proves indispensable in bioinformatics.

**Fig. 5.**
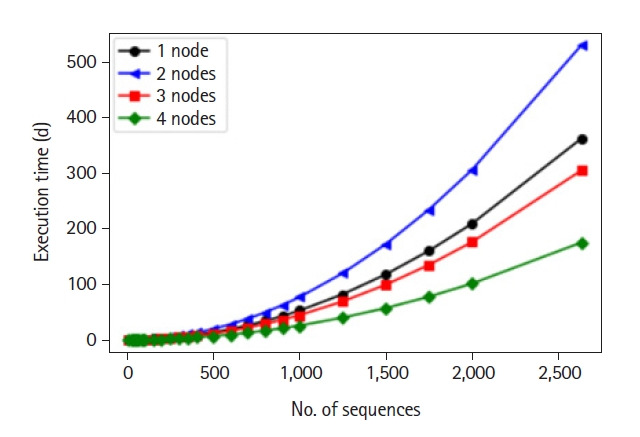
Estimated execution time of longest common subsequence algorithm using different numbers of nodes.

Afterwards, we deployed our LCS algorithm on a Hadoop cluster of four data nodes. We compared the execution time on different portions of data using different numbers of data nodes to assess the performance and prove the importance of our HPC architecture. Execution times where the number of sequences ranges from 5 to 100 were computed experimentally. However, for number of sequences beyond 100, we estimated the execution time by computing the average time it takes to perform a single pairwise comparison for each number of nodes. Obtained results are depicted in Fig. 5.

**Fig. 6.**
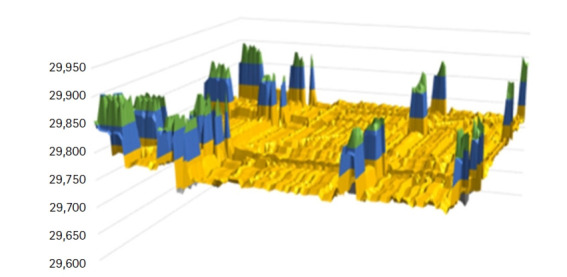
Range of the longest common subsequence (LCS) lengths. X and Y axis represent the sequences Z axis represent LCSs lengths.

As the data set size increases, the margin between the execution time using different nodes increases drastically. Thus, the more nodes we add to the cluster, the best performance we get. Besides showing the importance of using HPC to decrease execution time, the core of this work mandates using the results obtained from the deployment of the LCS algorithm to move to the next step of computing distance between all sequences. We constructed a 3D graph that visualizes the range of the length of the LCSs obtained for all sequence pairs, see Fig. 6.

X and Y axes correspond to all sequences constituting the dataset, and Z axis corresponds to the length of the LCS.

##### Distance/dissimilarity measuring

**Fig. 7.**
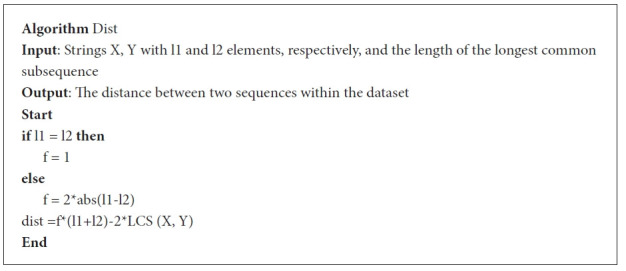
Dist algorithm.

In order to measure dissimilarity between sequences, we are proposing an algorithm that is based on the LCS algorithm output along with the lengths of both sequences (Fig. 7).

The idea is to have a positive integer as a measure that approximates the addition (l1+l2) to (2*LCS (X, Y)) whenever a pair of sequences are similar. l1+l2 (the addition of a pair of sequences lengths in the dataset) would always be greater or equal to two times the length of the LCS. The coefficient f is set to be greater or equal to 1 depending on the lengths of both sequences, f*(l1+l2) gets high whenever the value | len(seq1)-len(seq2) | (1) is high. We multiply (1) by 2 to make it more distant for unsimilar sequences. This formula considers the difference of sequences lengths to have an impact on the resulting output. However, we can get rid of the 2* operation used to get the coefficient f but it would be harder to detect the similarities since sequences would not be distant enough to perform appropriate clustering according to the formula.

Based on the proposed algorithm, sequences with the least distance correspond to those that are the most similar to each other. We are using the output of the distance algorithm to cluster all the sequences of our dataset. The results of the algorithm are arranged in a 2D matrix containing dissimilarity values between all pair of sequences constituting the dataset. The 2D matrix is afterwards used as input to the clustering algorithm, which is presented in the next section.

##### Clustering

Sequence clustering algorithms are used in bioinformatics to group biological sequences that are somehow related. Some clustering algorithms use single-linkage clustering, constructing a transitive closure of sequences with a similarity over a particular threshold.

UCLUST [29] and CD-HIT [30] are the most used algorithms to cluster amino acid sequences and using single-linkage clustering approach. They employ a greedy algorithm that finds a representative sequence for each cluster and assigns a new sequence to that cluster if it is sufficiently close to the representative; if no match is found, the sequence becomes the representative sequence for a new cluster. The alignment of sequences is often used to calculate the similarity score. For these algorithms, the threshold value is ambiguous.

A better alternative to avoid uncertainty of the threshold value is through UPGMA (unweighted pair group method with arithmetic mean) [31], which is a simple agglomerative hierarchical clustering method intended for the construction of rooted trees that reflects the structure in a matrix of pairwise similarity (or a dissimilarity) matrix. First, each sequence is considered as a cluster. The closest two clusters are combined into a higher-level cluster at each step until only one cluster remains. Clustering is done simply by taking the dissimilarity matrix as input and at each step, the pair of sequences A and B corresponding to the lowest dissimilarity value are combined to form a new cluster. Then, distances between the newly formed cluster and all other sequences must be updated to become the mean of both distances Dist(A, X) and Dist(B, X)

**Fig. 8.**
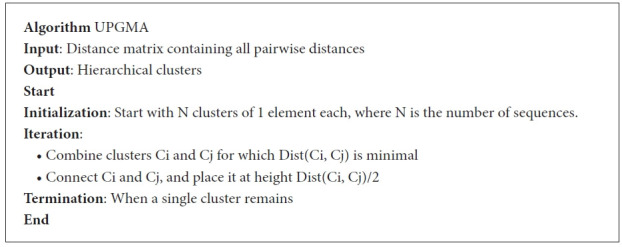
UPGMA (unweighted pair group method with arithmetic mean) algorithm.

For a better understanding of the UPGMA algorithm, we delineate it (Fig. 8).

##### Assessment

In order to assess the performance of the whole proposed methodology used to cluster SARS-CoV-2 sequences, the geolocation property as well as the identifier of each sequence are taken into consideration to visualize the tree resulting in the clustering step. The results are visualized in form of a large dendrogram, that we made available online [10].

As shown in the dendrogram, sequences coming from near geolocations are clustered together. Therefore, our proposed approach is accurate and we believe it will work fine in other applications of bioinformatics where no information about amino acids sequences is known and the studied popularity structure is mutating from an individual to another individual.

#### DS step 5: action

This concerns specialists in medicine and virologists in particular. In here, we presented the blueprint for an open source HPC and LCS-based platform for SARS RNA clustering. This can help researchers in medicine and virologists in clustering, categorizing, and thus identifying and tracking the rapid variance in SARS-CoV-2. Thus, contributing to limiting its widespread and threat.

All over the world, SARS-CoV-2 has impacted our daily lives to unexpected levels. All efforts need to be annexed towards understanding the evolution of the virus. To achieve this, ICT through HPC proves to be indispensable especially that the SARS-CoV-2 data falls within the realm of Big Data. The latter mandated the use of HPC. By presenting and promoting open source and cheap solutions, scientific communities all over the world can make their contribution towards limiting, and even stopping, the virus impact.

## Conclusion

In this paper, we presented the first phase we see needed to demystify SARS-CoV-2 unpredictable mutations, that consists of clustering/regrouping similar sequences, i.e., sequences presenting no important mutations that eventually form a putative SARS-CoV-2 type or sub-type. The resulting clusters can further get interpreted in future works aside with putative biological factors to accurately demystify the way the virus mutates and possibly predict the structure of the virus resulting when a host A affects a person B using appropriate machine learning model.

We first presented the algorithms and tools mostly used in bioinformatics to measure similarity between genomic sequences. We presented a DS approach aiming to extract value from our collected SARS-CoV-2 RNA sequences. We discussed and presented how HPC proves to be a better alternative for addressing slow execution problems faced in bioinformatics. Besides, we listed involved steps in transforming raw SARS-CoV-2 sequences into valuable information, i.e., the putative existing variations of the virus. We computed the execution time it takes to perform pairwise sequences comparisons in an ordinary computer and compared it to using HPC clusters with different number of nodes.

By presenting an open source and affordable cluster based HPC platform, we are contributing, and thus encouraging, the research community to use and adapt it to their typical needs. Having this said, the ultimate goal is to contribute to understanding and tracking SARS-CoV-2 unpredictable mutations and limit its widespread and impact on humanity.

## Figures and Tables

**Table 1.** Execution time of the comparisons of different portions of data using an ordinary laptop

No. of sequences	No. of comparisons	Execution time (min)
5	10	2
10	45	6
15	105	14
20	190	26
25	300	41
30	435	59

Foot notes
